# Using Palliative Leaders in Facilities to Transform Care for People with Alzheimer’s Disease (UPLIFT-AD): protocol of a palliative care clinical trial in nursing homes

**DOI:** 10.1186/s12904-023-01226-0

**Published:** 2023-07-26

**Authors:** Kathleen T. Unroe, Mary Ersek, Wanzhu Tu, Alexander Floyd, Todd Becker, Jessica Trimmer, Jodi Lamie, John Cagle

**Affiliations:** 1 Indiana University School of Medicine, Indianapolis, IN 46202 USA; 2 Regenstrief Institute, Inc, Indianapolis, IN 46202 USA; 3 University of Pennsylvania School of Nursing, Philadelphia, PA 19104 USA; 4 Department of Biostatistics, Indiana University, Indianapolis, IN 46202 USA; 5 University of Maryland School of Social Work, Baltimore, MD 21201 USA

**Keywords:** Nursing home, Palliative care, Dementia

## Abstract

**Background:**

Palliative care is an effective model of care focused on maximizing quality of life and relieving the suffering of people with serious illnesses, including dementia. Evidence shows that many people receiving care in nursing homes are eligible for and would benefit from palliative care services. Yet, palliative care is not consistently available in nursing home settings. There is a need to test pragmatic strategies to implement palliative care programs in nursing homes.

**Methods/design:**

The UPLIFT-AD (Utilizing Palliative Leaders in Facilities to Transform care for people with Alzheimer’s Disease) study is a pragmatic stepped wedge trial in 16 nursing homes in Maryland and Indiana, testing the effectiveness of the intervention while assessing its implementation. The proposed intervention is a palliative care program, including 1) training at least two facility staff as Palliative Care Leads, 2) training for all staff in general principles of palliative care, 3) structured screening for palliative care needs, and 4) on-site specialty palliative care consultations for a one-year intervention period. All residents with at least moderate cognitive impairment, present in the facility for at least 30 days, and not on hospice at baseline are considered eligible. Opt-out consent is obtained from legal decision-makers. Outcome assessments measuring symptoms and quality of care are obtained from staff and family proxy respondents at four time points: pre-implementation (baseline), six months after implementation, at 12 months (conclusion of implementation), and six months after the end of implementation. Palliative care attitudes and practices are assessed through surveys of frontline nursing home staff both pre- and post-implementation. Qualitative and quantitative implementation data, including fidelity assessments and interviews with Palliative Care Leads, are also collected. The study will follow the Declaration of Helsinki.

**Discussion:**

This trial assesses the implementation and effectiveness of a robust palliative care intervention for residents with moderate-to-advanced cognitive impairment in 16 diverse nursing homes. The intervention represents an innovative, pragmatic approach that includes both internal capacity-building of frontline nursing home staff, and support from external palliative care specialty consultants.

**Trial registration:**

The project is registered on ClinicalTrials.gov: NCT04520698.

**Supplementary Information:**

The online version contains supplementary material available at 10.1186/s12904-023-01226-0.

## Background

Nursing homes (NH) are an important site of care for people with dementia, particularly near the end of life. A substantial majority of NH residents (72%) have cognitive impairment, and half have a formal diagnosis of dementia [1, 2]. By 2030, an estimated 40% of all U.S. deaths among older Americans will occur in NHs, and 70% of people with advanced dementia will live their final days in a NH [3]. Unfortunately, NH care is associated with inadequate symptom control, low family satisfaction, burdensome treatments, and poor quality of care at the end of life [4–6]. Palliative care (PC) is an effective patient- and family-centered model of care focused on maximizing quality of life and relieving the suffering of people with serious illnesses. Studies have indicated that PC can improve NH resident outcomes, demonstrating better pain management, lower re-hospitalization, greater family satisfaction, and higher likelihood of receiving treatments consistent with one’s goals of care [7–11]. Experts have called for PC to be integrated as a standard model of care for all long-stay NH residents, but only isolated examples of successful implementation exist [12–15]. Despite evidence that PC could enhance NH care, PC remains highly underutilized, particularly among residents with dementia [8, 16–21]. In this manuscript, we describe a clinical trial to test the implementation and effectiveness of one such intervention.

## Project overview

This clinical trial is an evidence-informed intervention that provides internal capacity-building strategies for increasing PC knowledge and practice in NHs, as well as external specialty PC support to enhance the overall quality of care and quality of life for residents with dementia*.* The UPLIFT-AD (Utilizing Palliative Leaders in Facilities to Transform care for people with Alzheimer’s Disease) intervention includes training in-house PC Leads on key topics in PC and the Lead role, facility-wide PC overview education for staff, a structured process for screening for PC needs, and external specialty PC clinicians providing consultations in the facility. This federally funded clinical trial (NCT04520698) tests the effectiveness of the intervention, while assessing the implementation context [22]. The trial will include 16 NHs distributed evenly between Indiana and Maryland, with a target enrollment of 640 residents with moderate-to-advanced cognitive impairment. Enrollment of residents began in September 2021 and the first two facilities entered the implementation phase in January 2022. Final data collection for all facilities is expected to conclude in early 2025.

Collected via structured surveys, trial outcomes include changes in 1) resident symptoms and 2) perceptions of quality of care. Given the cognitive limitations of the study population, outcome data are collected from proxy respondents: NH staff and family/surrogate decision-makers for each resident. Secondary outcomes include NH staff knowledge and attitudes toward palliative care, collected at baseline and six months post-implementation.

**Fig. 1:**
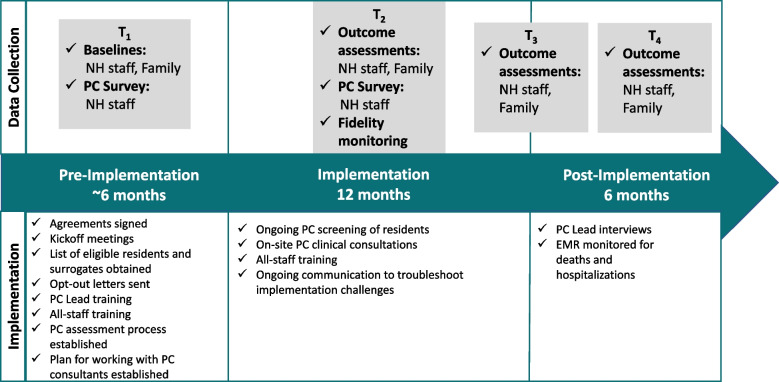
Timeline of activities in each nursing home

After recruitment of a NH, there is a pre-implementation period of up to six months, during which participants are enrolled, PC Leads are identified and trained, and baseline data are collected. The one-year intervention period begins when the first on-site visit from the external specialty PC consultant occurs. Follow-up data collection occurs six months after implementation and at the conclusion of implementation. The fourth and final data collection time point occurs six months following the end of the implementation period. Figure 1 shows the timeline for each NH, including data collection time points.

## Methods and design

### Study design and setting

UPLIFT-AD is a pragmatic trial with a modified stepped wedge design, where the intervention is rolled out in four waves. Each wave includes four facilities (two each in Indiana and Maryland). The sequence of the intervention rollout at the participating sites, however, is not fully randomized as in the traditional stepped wedge design to accommodate the logistics of rollout at specific NHs. Such ad-hoc modifications have been used in previous NH-based pragmatic trials [23]. In Indiana, the first four facilities were recruited and randomized to Waves 1 and 2. Further randomization of facilities, however, has proven too challenging, due to the many factors influencing successful NH recruitment. After discussion with the project Data Safety and Monitoring Board, the design was modified from a randomized to a non-randomized stepped wedge trial.

Eligible NHs must be within a 40-mile radius of the respective PC consultant practice in Indiana and Maryland (there are > 100 NHs in that radius for each state). NHs must be Medicare and Medicaid-certified and have a significant long-stay census, with a goal of having 40 residents who would meet inclusion criteria for the trial. NHs are excluded if they already have external PC consultants who make routine resident visits in the facility or if the facility has an active internal PC program.

### Objectives

The study will 1) evaluate the effectiveness of the UPLIFT-AD implementation by comparing surrogate-reported symptom management and quality of care of residents with dementia in intervention and control periods using structured outcome assessments; 2) evaluate the implementation of UPLIFT-AD using the RE-AIM (Reach, Effectiveness, Adoption, Implementation, and Maintenance) framework; and 3) compare staff knowledge and attitudes about PC, using a validated survey tool.

### Recruitment of nursing homes

Eligible facilities are contacted for additional information, including assessing whether organizational priorities are in alignment with PC services and the degree of interest in the study of key leaders. The research team and PC consultation partners conduct outreach to corporate contacts as well as facility-level leadership, including the facility administrator, director of nursing, and medical director, during the recruitment process. The facility administration signs a Research Collaboration Agreement prior to their involvement in the trial.

Participating NHs receive financial support ($15,000) to offset costs for their participation/partnership in the project (including staff time for data collection and trainings) and to facilitate the initial development of a sustainable PC program. To receive the financial stipend, project milestones must be met. The initial stipend of $10,000 is invoiced following signed agreements, initial in-facility kick-off meeting, and identification of PC Leads. The final $5,000 is invoiced following final data collection.

### Research ethics

The UPLIFT-AD protocol is approved by the Indiana University Institutional Review Board and follows recommendations for intervention trials according to the SPIRIT checklist [24]. The Research Collaboration Agreement signed by NH administrators and Indiana University includes data privacy and protection provisions. Study data are entered into a secure REDCap database to which only study personnel have access.

Potentially eligible residents are identified by the NH leadership, with eligibility confirmed by research staff. A study information sheet (see Additional File 1) and consent form, with opt-out instructions (see Additional File 2), are mailed to the surrogate decision-maker for each resident. Surrogate decision-makers can opt the resident out of the study via phone, email, or REDCap link. An opt-out consent process for residents has been determined to be appropriate for this minimal-risk study. Consent for treatment must be obtained separately prior to a PC clinical consult taking place.

For outcome assessments, staff members who are familiar with the health and care of the enrolled residents over the past 30 days are identified by supervisors or by asking staff directly which residents they know best. Research staff, presenting the study information sheet (see Additional File 3) then approach staff to enroll them in the study and collect outcome data. A staff member may provide outcome data for more than one resident. Documented verbal consent by the staff member is obtained at the time of enrollment. In addition, research staff enroll surrogate decision-makers, typically family members, for outcome assessments. Surrogate contact information is obtained from the NH. Verbal consent is obtained and documented prior to surrogate participation in outcome assessments.

Frontline clinical staff (e.g., nurses and nursing assistants) are offered the Palliative Care Survey [25–27], which includes a study information sheet that states that completion of the survey indicates consent for data to be analyzed.

### Description of the intervention

#### PC Lead role

In-house PC Leads (minimum of 2 per facility, at least one RN or LPN) are existing NH staff members identified to champion NH-based delivery of the intervention. The research team encourages interdisciplinary representation among the PC leads, which may include nurses, social services providers, or chaplains, based on the roles and existing skill sets within the facility. The involvement of nursing is considered key for symptom assessment and management. The UPLIFT-AD research team developed a specialized training framework for leads, which includes approximately eight hours of training content delivered by an experienced PC clinician educator that covers four core modules: An overview of PC in the NH; team and family communication, including content on dementia-specific advance care planning approaches; pain and symptom management; and emotional support and self-care (see Table 1). Leads are also trained to conduct PC assessments, implement strategies to address common symptom/psychosocial/spiritual needs, support residents’ families, facilitate case reviews, and make referrals to the PC consultants. Lead education is designed to be delivered either in-person or virtual, depending on facility and staff preferences.

**Table 1:** Palliative care lead education topics

• Overview of UPLIFT
• Resident-Centered Dementia Care
• Palliative and End-of-Life Care
• Communication Part 1: Fundamentals
• Communication Part 2: Advance Care Planning
• Promoting Resident Quality of Life
• Common Distressing Symptoms Part 1: Pain
• Common Distressing Symptoms Part 2: Other Symptoms
• Palliative Care Assessment and Screening
• Taking Care of Yourself
• Ethical, Legal, Financial, and Cultural Considerations

#### All staff education

Providing education on PC to all staff interacting with UPLIFT-AD residents in the facility is a core element of the UPLIFT-AD intervention. Experienced clinical educators from the research team and the external specialty PC consultants—all with extensive expertise in geriatrics and PC—collaborate on delivering education. Key topics include an overview of PC, objectives of the UPLIFT-AD project, resident-centered dementia care, and communication with families. These sessions are delivered as part of all-staff meetings, as well as smaller group sessions which may be held in “huddles” in clinical areas. Additional topics are added upon request from staff, for example, managing difficult dementia-related behavior.

#### PC screening

A key challenge for PC specialty providers is identifying residents appropriate for clinical consults. All participating facilities are implementing a structured process for resident PC needs assessment. PC Leads complete these assessments and determine whether there is a clinical need for PC consultation. The screening tool (see Additional File 4) was developed by the research team, with input from clinical PC partners. It includes absence of, or conflict around, goals of care documentation; poorly managed symptoms; recent hospitalization; polypharmacy; significant functional decline; or other PC needs identified by a PC Lead or other clinical provider.

### Palliative care consultations

PC consultants from a hospital-based PC practice (Indiana) and a large community-based hospice and PC organization (Maryland) are providing on-site consultation visits with residents during the implementation period. Project funds are dedicated to the PC consultant team for a registered nurse’s (RN) or nurse practitioner’s (NP) time to support the capacity building needed to coordinate and deliver specialty care to residents. Consultants (MDs and NPs) can also bill Medicare Part B for billable clinical encounters.

Using the PC screening tool, PC Leads support PC consultants in identifying residents for clinical consults. PC consultants have a consistent schedule in the facility (e.g., ½ day per week or a full day twice monthly). When hospice services are initiated for a resident in the intervention, the in-house PC Leads continue to provide supportive services to the resident and family; however, the PC consultant team transfers clinical care to the hospice providers.

### Flexible and core elements

Nursing homes are dynamic clinical environments and often experience challenges in recruiting and maintaining a well-prepared workforce. As a result, staff often have multiple roles, such as supporting new admissions, scheduled tasks and assessments, routine care, and responding to acute changes in status. Staff and administrator turnover is also common [28]. The research team has extensive experience working in and implementing programs in this setting and familiarity with the reality of complex clinical workflows and stressors. The UPLIFT-AD intervention was designed to optimize implementation through incorporating core elements, as well as built-in flexibility for other elements (see Table 2).

**Table 2:** UPLIFT intervention elements

**Core Intervention Elements**	**Flexible Intervention Elements**
• External PC consultations on-site	• Visit schedule
• In-house PC Leads	• Job role of the leads – RN, LPN, social services, or chaplain
• NH staff participation in education	• Schedule of education topics and format of delivery
• Process for PC assessments	• Meeting schedules

### Implementation strategies

The UPLIFT-AD trial includes multiple structured implementation processes. After agreements are signed by corporate and facility level leadership, the kick-off meeting is scheduled. This meeting is attended by NH administrative and clinical leadership, social services, and, when possible, medical providers. The overall rationale and goals of the project are shared, as well as a description of the PC Lead role (see Additional File 5), education topics, PC screening process, and role of PC specialty consultants. A timeline, including detailed description of subject enrollment and data collection, is shared.

Ongoing check-in meetings with research staff and PC Leads throughout the implementation phase allow for monitoring of fidelity to the intervention. These meetings are initially held weekly and then may shift to monthly based on the PC Lead preference after the first month if there is consistent engagement and responsiveness to the research team. During these check-ins, the eligible resident list is reviewed for changes, PC screening status is reviewed, and any troubleshooting regarding PC consults or other issues is discussed.

Any concerns with engagement are generally resolved through communication with the PC Leads or facility-level leadership. The research team also identifies corporate-level contacts for cases when additional support is needed, e.g., IT support to navigate receiving secure emails at the facility for project communications.

Another critical aspect of implementation that requires initial and ongoing management is the communication of recommendations resulting from PC specialty consultations. Recommendations from consultants must be communicated to primary care providers in the facility. Thus, preferences for communication (e.g., use of a folder at the facility; emailing clinical notes) are established upfront and reviewed regularly. A key aspect of the PC Lead role is following up on recommendations from the clinical PC consultants.

### Screening and enrollment

**Fig. 2:**
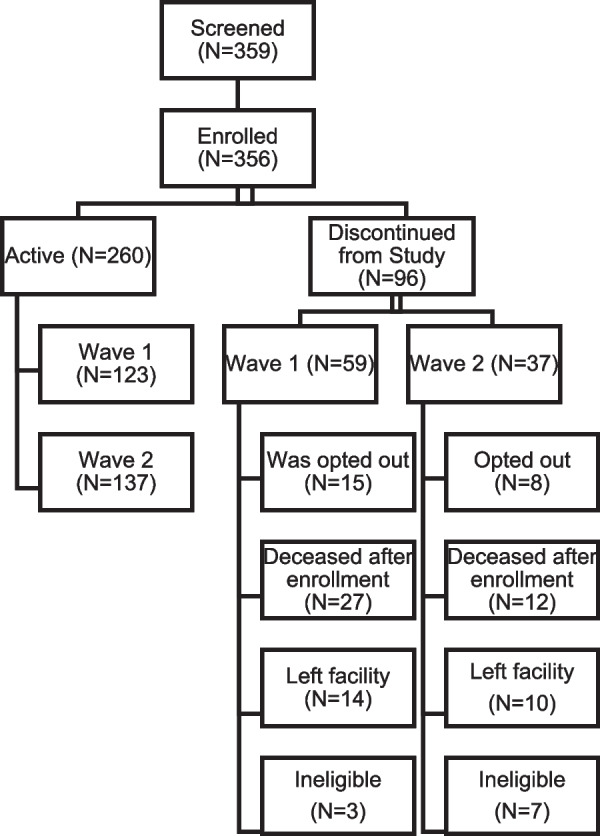
Overall Patient Study Status for First 8 Facilities (Target *N* = 320), Recruitment start date: 9/1/21. Data current through 7/25/2022

Potentially eligible NH residents are identified and screened by study staff by reviewing a list of potentially eligible residents identified by NH representatives. After NH representatives run a report of the BIMS (Brief Inventory of Mental Status) scores of all residents, the research team reviews eligibility criteria (detailed in the subsequent section) with NH staff for all residents with a BIMS score of 12 or less. All eligible residents’ surrogate decision-makers receive an opt-out letter for resident participation in the study. In accordance with the IRB-approved protocol, if no opt-out response is received within one week of mailing, the residents are then enrolled in the study. The research team collects data from NH staff on all UPLIFT-enrolled residents for the primary study outcomes. Surrogate decision-makers are contacted by phone to enroll them in the study for the purposes of data collection. See Fig. 2 for recruitment data regarding the first 8 facilities through 7/25/22.

### Inclusion/exclusion criteria

There are three types of research participants in this study: 1) NH residents with indications of cognitive impairment; 2) staff who care for enrolled residents; and 3) surrogate decision-makers (usually family members) of enrolled residents. Inclusion criteria for NH residents are 1) in the facility > 30 days; 2) not currently on Medicare Part A funded rehabilitation care; 3) not receiving hospice services at time of enrollment; and 4) moderate-to-advanced cognitive impairment, with a BIMS score of 12 or less on the most recent assessment prior to enrollment. Inclusion criteria for staff are 1) identified by a supervisor as a person who regularly cares for the resident; 2) employee of the facility; and 3) English-speaking. Inclusion criteria for surrogate decision-makers or family members of residents are 1) familiarity with the care of the resident; and 2) English-speaking. Only one staff member and one surrogate decision-maker provide data for any given resident at each time period; different staff or family members may be enrolled and provide data at later time points for a given resident if needed.

NH staff are recruited to complete Palliative Care Surveys at baseline and six months after implementation. All nursing assistants and licensed nurses (i.e., RNs and LPNs) who interact with UPLIFT-AD residents in the facility are eligible to complete the Palliative Care Survey.

### Measures

The goal of the study is to demonstrate impact of a PC program implementation for NH residents with moderate-to-advanced cognitive impairment using several validated measures. The EOLD-CAD (End of Life Dementia – Comfort Assessment in Dying) scale is designed for proxy report of symptoms, including pain, shortness of breath, restlessness, and calm. It has been used in studies evaluating care for residents with advanced dementia in NHs and as an outcome measure in PC studies [29–34]. The EOLD-SM (End of Life Dementia – Symptom Management), quantifies the frequency of nine signs or symptoms, including pain, shortness of breath, depression, fear, anxiety, and agitation [31]. For the UPLIFT-AD study, the outcome assessment instrument includes the modified EOLD-CAD and EOLD-SM items and measures proxy report (for both staff assessments and surrogate decision-maker/family assessments) of the frequency and intensity of symptoms over the past month. In addition, family respondents are asked questions from the EOLD-SWC (End of Life Dementia – Satisfaction with Care) scale [31, 35]. Table 3 (pp. 25–26) summarizes information about all study measures.

**Table 3:** Key study measures for the UPLIFT-AD trial by study aim

Measure	Construct	Description	Properties (subscales & reliability coefficients if appropriate)	UPLIFT Data Source(s)	UPLIFT Aim	Data Collection Periods
EOLD-CAD	Intensity of symptoms and conditions	*Items*: 14 *Recall*: Past 30 days *Response options*: 3-point ranging from “not at all intense” to “very intense”^a^	Full scale [36]: α = .85 Subscales [36]: Physical Distress: α = .74 Emotional Distress: α = .82 Well Being: α = .80 Dying Symptoms: α = .70	NH Staff Family	Aim 1	T1, T2, T3, T4
EOLD-SM	Frequency of physical and emotional symptoms	*Items*: 9 *Recall*: Past 30 days *Response options*: 6-point ranging from “never” to “every day”	Full scale [36]: α = .78 Subscales [36]: Psychological Symptoms: α = .81 Physical Symptoms: α = .47^b^	NH Staff Family	Aim 1	T1, T2, T3, T4
EOLD-SWC	Satisfaction with care	*Items*: 10 *Recall*: Past 30 days *Response options*: 4-point ranging from “strongly agree” to “strongly disagree”	Full scale [36]: α = .90 Subscales: [None]	Family	Aim 1	T1, T2, T3, T4
Palliative Care Screen	Assessment of resident PC needs; acuity assessment	*Items*: 6 *Recall*: Present *Response options*: Yes/No	N/A; created by the UPLIFT research team; items cover non-concordant goals of care; lack of ACP documentation; hospitalization; unmanaged symptoms; polypharmacy; complex care	PC Leads	Aim 2	Within 1 month of UPLIFT initiation; then as needed
Fidelity Checklist	UPLIFT Implementation Tracking	5 domains	N/A; created by the UPLIFT research team; domains include referrals/consultations; hospitalizations; deaths; adverse events; change in PC needs	PC Leads and/or PC Consultants	Aim 2	At least monthly during UPLIFT implementation
Debriefing Interviews	Qualitative perceptions of implementation	Semi-structured interviews	N/A; created by the UPLIFT research team; includes perceptions of training, areas for improvement, sustainability	PC Leads	Aim 2	T3
Palliative Care Survey	Knowledge of and engagement in PC practices	*Items*: 51 *Recall*: Varied *Response options*: Varied	Subscales [26]: PC Practice Subscale: α = .75 PC Knowledge Subscale: α = .81	NH Staff	Aim 3	T1, T2

“Family” respondents are broadly defined as the person responsible for making health-related decisions on behalf of the UPLIFT resident, which occasionally includes guardians, or close friends

T1 = Baseline; T2 = 6 months post-UPLIFT initiation; T3 = 1 year post-UPLIFT initiation; T4 = 1.5 years post-UPLIFT initiation (i.e., 6 months after UPLIFT discontinuation)

*NH* Nursing home, *PC* Palliative care, *ACP* Advance Care Planning, *EOLD-CAD* End of Life Dementia – Comfort Assessment in Dying scale, *EOLD-SM* End of Life Dementia—Symptom Management scale, *EOLD-SWC* (End of Life Dementia – Satisfaction with Care) scale

^a^The original EOLD-CAD response option indicating “a lot intense” was modified to instead read “very intense” during pilot testing of study measures during

^b^Due to the low correlation among Physical Symptom items (*n* = 3), the scale creators suggest assessing these three items independently

In order to evaluate the impact on the education program included in UPLIFT-AD and the diffusion of knowledge from the presence of a PC consult program, a Palliative Care Survey is conducted in the NHs at baseline and six months post-implementation. Multiple frontline clinical staff are asked to complete the survey. The Palliative Care Survey is a validated instrument to measure NH staff knowledge, attitudes, and practices regarding PC [25–27]. It includes patient vignettes and questions regarding frequency of key behaviors, such as family communication.

### Statistical analyses

The primary outcome of the trial is the EOLD-CAD as assessed by NH staff. Family-reported symptom data will be analyzed separately and compared to staff measures for agreement. Analysis will be carried out in an intention-to-treat framework [37]. Consistent with the analysis of stepped wedge trials, we will implement the analysis in mixed-effect models [38]. A key independent variable to be included is the binary indicator for treatment periods (0 = pre-intervention, 1 = post-intervention). Other resident characteristics, such as age, sex, race, and degree of cognitive and functional impairment, will be modeled as covariates. As in trials with a stepped wedge design, all time-invariant resident and facility characteristics will be balanced between the control and intervention periods. Time-varying resident characteristics, however, may be unbalanced. The influences of these factors will be controlled for in the mixed-effects model analysis. Facility-specific random intercepts will be included to accommodate the potential correlations in the outcomes contributed by residents from the same facility. All analyses will be conducted in SAS. The main hypotheses will be tested through PROC MIXED, using a two-sided Wald test. *P* values < 0.05 will be considered statistically significant.

Using the Palliative Care Survey, linear mixed-effects models will be used to compare NH staff PC knowledge and attitudes over time and between conditions. Scores from respondents in the same NH are expected to be correlated; thus, facilities will be included in the model as a random effect. Factors in each model will include respondent and facility characteristics.

### Power calculation and sample size

With a projected sample size of 640 UPLIFT-AD residents (an average of 40 enrolled residents per facility), we have > 90% power to detect an intervention effect size of 0.5 standard deviations in the primary outcome, EOL-CAD, when the level of intraclass correlation is between 0.1 and 0.2. Van der Maaden’s recent trial observed an ICC of 0.10 [39]. An effect size of 0.5 typically indicates a medium level of intervention effect [40, 41]. For the outcomes of interest,

Volicer and colleagues reported that the standard deviations of the EOLD-CAD and EOLD-SWC from a similar population were ~ 6 [36]. An effect size of 0.5, therefore, assures ample power for detecting a change of 3 points in EOLD-CAD and EOLD-SWC. Similarly, for the Palliative Care Survey completed by NH staff, the study will also have sufficient power to detect a difference of 0.5 standard deviations [26]. Based on our experience, intervention effects of such magnitudes are of clinical significance.

### Implementation evaluation

Within the RE-AIM [42–44] framework, Reach is defined as the proportion of the target population who participate in the intervention. We will assess the percentage of eligible residents with dementia who receive at least one PC assessment. We anticipate that > 75% of enrolled residents will receive a PC assessment. Effectiveness will be assessed by the primary study outcome. Adoption is defined as the degree to which PC Leads implement the program as intended, as assessed from regularly scheduled check-in reports and qualitatively through PC Lead interview data. Implementation will be tracked by ongoing fidelity monitoring and assessment of clinical notes from PC consultations. Maintenance includes ongoing contact with the research team to encourage adherence to the protocol and to maintain productive relationships with PC consultants during the implementation period.

## Discussion

The UPLIFT-AD intervention is designed to address the challenge of meeting the PC needs of residents in NHs in a pragmatic fashion, with the goal of creating and testing a replicable model of care. The UPLIFT-AD trial began recruitment of NHs in mid-2021. NH recruitment and participant recruitment is ongoing and final data collection is anticipated to occur in early 2025.

The launch and early implementation of this project included multiple challenges. Some of these challenges were anticipated when the project was originally conceptualized, such as NH staff turnover. To mitigate interruptions in the intervention due to turnover, we are recruiting and training a minimum of two PC Leads at each site and offering ongoing access to staff PC education. Given the long experience of the research team in working in the NH clinical setting, another challenge that was anticipated is the difficulty in integrating new clinical providers into this complex, reactive healthcare setting. An explicit aspect of the PC Lead role is to serve as an anchor point for the external PC consultants; they support the implementation of recommendations and communicate information across clinical providers and other care staff. The PC Consultants also invest time building relationships with the clinical leadership (e.g. primary care providers) in the facility and elicit communication preferences.

Other challenges were not anticipated when the study was designed, such as COVID-19 outbreaks and the degree of instability in the industry, including shifts in corporate ownership. This has caused difficulties in recruiting facilities. NH leaders, while generally supportive of PC and the support it may offer residents, were unable to commit to a project with an uncertain start date, thus we were unable to randomize all facilities in terms of start dates in the original stepped wedge design. Instead of having all 16 NHs recruited and randomized at the start of the study as planned, we assigned the sequence of intervention rollout according to the availability of NHs that expressed interest in participating. This accommodation was considered necessary to address the preferences of the NH providers.

This article describes the protocol for implementing a multi-component PC model in a stepped wedge trial in 16 NHs. Interventions and programs designed to improve the care experience of residents all involve adoption of new or refinement of existing clinical practices. A large review describing NH interventions that were designed to change staff practices identified that interventions with theory-based implementation strategies, designed to address common barriers, had stronger impact [45]. The UPLIFT-AD intervention, led by a team with deep experience in both clinical practice and research in the NH setting, includes multiple flexible implementation elements. If successful, UPLIFT-AD may serve as a standard for PC delivery in NHs.

## Supplementary Information

**Additional file 1. ***UPLIFT Family One-Pager.docx*. A one page UPLIFT information sheet mailed to family of UPLIFT-qualifying residents, as identified by NH leadership and confirmed by research staff.

**Additional file 2.** *UPLIFT Opt-Out Letter.docx*. A description of UPLIFT and opt-out directions for residents, mailed to family of UPLIFT-qualifying residents, as identified by NH leadership and confirmed by research staff.

**Additional file 3. ***UPLIFT NH One-Pager.docx*. A one page UPLIFT information sheet presented to eligible UPLIFT staff prior to enrollment.

**Additional file 4. ***Palliative Care Screening Tool.docx*. A tool developed by the research team to screen NH residents for qualification for a PC consultation.

**Additional file 5. ***Palliative Care Lead Role.docx*. A description of the PC lead role and the responsibilities of the leads.

## Data Availability

The data that support the findings of this study are available from Centers for Medicare and Medicaid Services, but restrictions apply to the availability of these data, which were used under license for the current study, and so are not publicly available.

## Abbreviations

UPLIFT-AD: Utilizing Palliative Leaders in Facilities to Transform Care for Alzheimer’s Disease
NH: Nursing home
PC: Palliative care
RE-AIM: Reach, Effectiveness, Adoption, Implementation, and Maintenance Framework
RN: Registered nurse
NP: Nurse practitioner
LPN: Licensed practical nurse
BIMS: Brief Inventory of Mental Status
EOLD-CAD: End of Life Dementia – Comfort Assessment in Dying Scale
EOLD-SM: End of Life Dementia – Symptom Management Scale
EOLD-SWC: End of Life Dementia – Satisfaction with Care Scale
