# Supplementary material for: Using Palliative Leaders in Facilities to Transform Care for People with Alzheimer’s Disease (UPLIFT-AD): protocol of a palliative care clinical trial in nursing homes
Source: BMC Palliat Care. 2023 Jul 26;22:105. doi: 10.1186/s12904-023-01226-0 (PMC10369841; doi:10.1186/s12904-023-01226-0)
Supplement: Supplementary file 1 — Additional file 1. UPLIFT Family One-Pager.docx. A one page UPLIFT information sheet mailed to family of UPLIFT-qualifying residents, as identified by NH leadership and confirmed by research staff. [file 12904_2023_1226_MOESM1_ESM.docx]

| 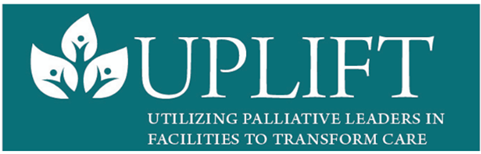 | | | |
| --- | --- | --- | --- |
| What does UPLIFT address? | | How will UPLIFT improve care? | |
| - Research has shown that people with dementia could benefit from palliative care - Palliative care improves symptom management, reduces burdensome treatment, reduces hospital visits, and improves family satisfaction - About 70% of people with advanced dementia will live their final days in a nursing facility | | UPLIFT is a research-informed model of care  It improves palliative care by:  **Training** nursing home UPLIFT palliative champions  **Educating** nursing home staff on basic palliative care  **Facilitating access** to external palliative care experts  **Integrating** primary and specialty palliative care to provide **compassionate care** | |
| How does UPLIFT work? | | We need your help! | |
| **In-house palliative champions** — At least 2 nursing home staff members will receive structured training on:   - Palliative care, symptom management, dementia specific care   **External palliative care consultants** —   - [Palliative Care Partner]-based palliative care consult team - Will work with residents and their families to establish or clarify treatment goals and provide medical symptom management | | We will contact you as the surrogate decision maker to ask if you are interested in participating in the research. This will include data collection about the resident and surveys for you to complete. | |
| What are the benefits of participating? | | How are we addressing COVID-19 concerns? | |
| The advance care planning will likely prompt discussions regarding preferences, quality-of-life, and symptoms. Participation in these discussions may result in closer alignments between you and your family member’s care preferences and the treatments they receive | | - Any face to face visits will involve facility-based protective measures and CDC best practices - Most interactions, however, will be conducted via phone or video conferencing - Face to face interactions will be conducted by staff well-trained in nursing home COVID protocols | |
| **Our Study Team** | | **Who do I contact?** | |
| [Study team members] |  | **[University Name]:**  [Investigator and contact information]  [Coordinator and contact information]  **[University Name]:**  [Investigator and contact information]  [Coordinator and contact information] |  |
