# Supplementary material for: Using Palliative Leaders in Facilities to Transform Care for People with Alzheimer’s Disease (UPLIFT-AD): protocol of a palliative care clinical trial in nursing homes
Source: BMC Palliat Care. 2023 Jul 26;22:105. doi: 10.1186/s12904-023-01226-0 (PMC10369841; doi:10.1186/s12904-023-01226-0)
Supplement: Supplementary file 2 — Additional file 2. UPLIFT Opt-Out Letter.docx. A description of UPLIFT and opt-out directions for residents, mailed to family of UPLIFT-qualifying residents, as identified by NH leadership and confirmed by research staff. [file 12904_2023_1226_MOESM2_ESM.docx]

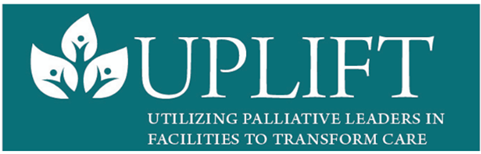


Dear [Family Member Name],

We are researchers at [University Name] and [University Name] leading a new project – UPLIFT. We have partnered with [Facility Name] nursing facility on this National Institutes of Health funded project. We will be supporting staff in the facility in providing the best possible care for residents with memory problems and dementia. Staff will receive additional training in palliative care, which is medical care for people with serious illness that focuses on symptoms and care coordination. Palliative care is delivered as an additional support, along with other medical treatments, and is considered a standard of care for anyone with serious illness. Ultimately, the goal of the program is to support care provided in nursing homes though education and bringing additional resources to the nursing home. A total of 16 nursing homes in [State] and [State] will participate in the project.

Residents are considered eligible for UPLIFT if they have been in the facility more than 30 days and have moderate to advanced memory problems or dementia. We would like to track data related to your family member for this project. No data will be collected directly from any resident. To participate, residents will have information about their health and the care they receive shared with the research team. Nursing facility staff will also answer brief surveys about residents’ health and the care they receive. In addition, you will be contacted to ask if you would like to participate in surveys related to this project. This data collection will involve questions about the resident’s experience with care in the nursing facility. It is up to you whether you directly participate in this data collection. This information will be kept private, in accordance with Federal and State privacy laws.

Starting in [Month Year], [Palliative Care Partner] palliative care providers will be available to provide consultations to residents if medically indicated. If the clinical team at the facility feels a palliative care consult would be helpful for your family member, you would be contacted prior to the visit. These providers may have discussions regarding the resident’s care, make recommendations to the nursing home to improve the resident’s comfort, or alleviate symptoms. There are no costs associated with participation in the UPLIFT project. However, [Palliative Care Partner] will bill insurance through standard means and there may be costs associated with services or medications provided. Again, this would be discussed prior to any medical consultation visit.

As the resident’s surrogate decision-maker you can choose for the resident not to participate in this project, including data collection or an opportunity for palliative care consultations. To decline participation, please click this link, [survey link] or scan the QR code below with your phone camera.

For more information about UPLIFT, please contact [Project Coordinator] at [Email] or [Phone].

[QR Code]

Sincerely,

[Investigator’s signature]

[Investigator’s name and title]
