# Supplementary material for: Using Palliative Leaders in Facilities to Transform Care for People with Alzheimer’s Disease (UPLIFT-AD): protocol of a palliative care clinical trial in nursing homes
Source: BMC Palliat Care. 2023 Jul 26;22:105. doi: 10.1186/s12904-023-01226-0 (PMC10369841; doi:10.1186/s12904-023-01226-0)
Supplement: Supplementary file 3 — Additional file 3. UPLIFT NH One-Pager.docx. A one page UPLIFT information sheet presented to eligible UPLIFT staff prior to enrollment. [file 12904_2023_1226_MOESM3_ESM.docx]

| 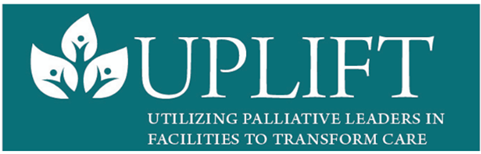 | | | |
| --- | --- | --- | --- |
| What does UPLIFT address? | | | How will UPLIFT improve care? |
| - Research has shown that people with dementia could benefit from palliative care - Palliative care improves symptom management, reduces burdensome treatment, reduces hospital transitions, and improves family satisfaction - About 70% of people with advanced dementia will live their final days in a nursing facility | | | - UPLIFT is an evidence-informed model of care - It improves palliative care by:   **Training** in-house UPLIFT palliative champions  **Educating** all staff on basic palliative care  **Facilitating access** to external palliative care experts  **Integrating** primary and specialty palliative care to provide **compassionate care** |
| How does UPLIFT work? | | | What do we expect from partner nursing homes? |
| **In-house palliative champions** — At least 2 clinical staff members will receive ~8 hours of structured training on:   - Palliative care, conducting palliative care assessments, facilitating advance care planning conversations, symptom management, training others in palliative care, dementia specific advanced care planning, and more   **External palliative care consultants** —   - [Palliative Care Partner]-based palliative care consult team - Will work with residents and their families to establish or clarify goals of care and provide medical symptom management - Providing an expected minimum of four consults, per facility, per week | | | We will ask staff who care for residents with dementia if they are interested in participating in research. This may include completing a series of questionnaires about palliative care. If you are involved in providing care to residents, you may be asked to complete questionnaires about their perceptions of their care and quality of life. Family members of residents with dementia will also be asked to complete questionnaires. |
| What are the benefits of participating? | | | How are we addressing COVID-19 concerns? |
| - Nursing facility residents with ADRD receive additional services focused on comfort, symptom management, and establishing and honoring care preferences - Training and education on palliative care – eligible for CEU credits - No additional costs - Engage in a true partnership and collaboration with experts compassionate about resident care | | | - We will initiate data collection and study procedures at a mutually agreed on time - Any face-to-face visits will involve facility-based protective measures and CDC best practices - Most interactions, however, will be conducted via phone or video conferencing - Face to face interactions will be conducted by staff well-trained in nursing home COVID protocols |
| **Our Study Team** | | **Who do I contact?** | |
| [Study team members] |  | | **[University Name]:**  [Investigator and contact information]  [Coordinator and contact information]  **[University Name]:**  [Investigator and contact information]  [Coordinator and contact information] |
