# Supplementary material for: Using Palliative Leaders in Facilities to Transform Care for People with Alzheimer’s Disease (UPLIFT-AD): protocol of a palliative care clinical trial in nursing homes
Source: BMC Palliat Care. 2023 Jul 26;22:105. doi: 10.1186/s12904-023-01226-0 (PMC10369841; doi:10.1186/s12904-023-01226-0)
Supplement: Supplementary file 4 — Additional file 4. Palliative Care Screening Tool.docx. A tool developed by the research team to screen NH residents for qualification for a PC consultation. [file 12904_2023_1226_MOESM4_ESM.docx]

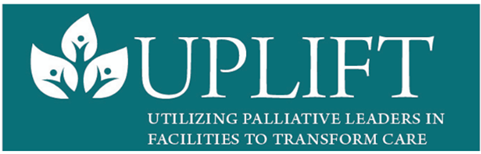


PALLIATIVE CARE SCREENING TOOL

Name _________________________ Date _______________________

Facility _________________________ Last assessment ______________

**Directions: If any screening items below are checked, a referral must be made to the UPLIFT consultant team.**

□ No goals of care or advance care planning documentation

□ Conflict around Goals of Care or treatment plan

□ Unmanaged symptoms such as pain, difficulty breathing, swallowing, or challenging behaviors

□ Hospitalization in the last 30 days

□ Polypharmacy – 9 or more medications (including PRN), or concerns around medications

□ Significant functional decline

□ Any other reason this resident may benefit from a palliative care consult

Notes ______________________________________________________________________________________________________________________________________________________________________________________________________________________________________________________________________________________________________

□ Palliative Care consult not indicated at this time

□ Palliative Care consult is indicated

□ Priority Level for when consult will be scheduled

Notes __________________________________________________________________________________________________________________________________________________________________________________________________________________________________________________________________________________________________________________

□ Routine (within a month)

□ Next available

**Referral Process:**

- Send the following documents to [Palliative Care Team Member]
  - Assessment sheet
  - ACP document (POST- both sides, DNR)
  - Face Sheet

Completed by _______________________________ Date __________________
