# Supplementary material for: Using Palliative Leaders in Facilities to Transform Care for People with Alzheimer’s Disease (UPLIFT-AD): protocol of a palliative care clinical trial in nursing homes
Source: BMC Palliat Care. 2023 Jul 26;22:105. doi: 10.1186/s12904-023-01226-0 (PMC10369841; doi:10.1186/s12904-023-01226-0)

| Description and Responsibilities of UPLIFT Leads |
| --- |
| - Conducting palliative care assessments   - Quarterly assessments for each resident, unless they have been seen in the last 30 days by palliative care - Initiating and following-up on ACP discussions   - Track new conversations, which include initial conversations, follow-up conversations, significant changes in condition, care conferences, any other time that goals of care are discussed - Placing palliative care assessments, advance care planning, and other encounters in medical record - Referring residents and families to palliative care consultants - Serve as a palliative care resource to other nursing home staff - Attending quarterly meetings with project team - Supporting data collection for research purposes - Reporting adverse reactions to palliative care conversations to UPLIFT staff within 1 business day - Follow up with residents and families to ensure care plans are implemented |


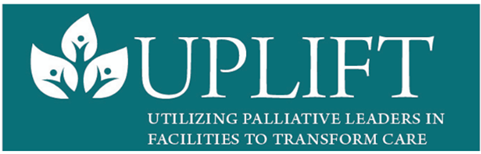

Supplement: Supplementary file 5 — Additional file 5. Palliative Care Lead Role.docx. A description of the PC lead role and the responsibilities of the leads. [file 12904_2023_1226_MOESM5_ESM.docx]
